# *cis*-[(7*R*,14*R*)-5,5,7,12,12,14-Hexa­methyl-1,4,8,11-tetra­azacyclo­tetra­decane-κ^4^ *N*](oxalato-κ^2^ *O*,*O*′)nickel(II) oxalic acid solvate

**DOI:** 10.1107/S1600536809020042

**Published:** 2009-06-06

**Authors:** Guang-Chuan Ou, Yong-Qiang Dai, Man-Sheng Tang

**Affiliations:** a Department of Biology and Chemistry, Hunan University of Science and Engineering, Yongzhou Hunan 425100, People’s Republic of China

## Abstract

Both mol­ecules of the title compound, [Ni(C_2_O_4_)(C_16_H_36_N_4_)]·C_2_H_2_O_4_, are located on a crystallographic twofold rotation axis. The Ni^II^ atom shows a distorted octa­hedral geometry. The crystal packing is stabilized by N—H⋯O and O—H⋯O hydrogen bonds.

## Related literature

For general background, see: Tait & Busch (1976 ▶); Curtis (1965 ▶). For a related crystal structure, see: Tang *et al.* (2002 ▶).
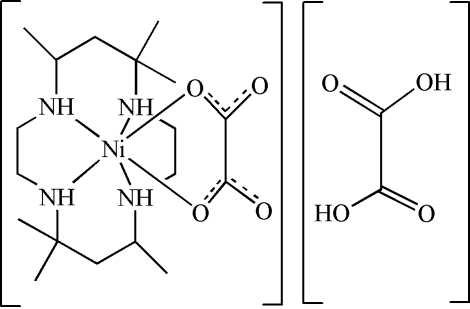


## Experimental

### 

#### Crystal data

[Ni(C_2_O_4_)(C_16_H_36_N_4_)]·C_2_H_2_O_4_
*M* *_r_* = 521.25
Orthorhombic, 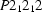

*a* = 10.1261 (15) Å
*b* = 15.515 (2) Å
*c* = 8.0467 (11) Å
*V* = 1264.2 (3) Å^3^
*Z* = 2
Mo *K*α radiation
μ = 0.82 mm^−1^
*T* = 173 K
0.48 × 0.21 × 0.15 mm

#### Data collection

Bruker SMART CCD area-detector diffractometer
Absorption correction: multi-scan (*SADABS*; Sheldrick, 1996 ▶) *T*_min_ = 0.695, *T*_max_ = 0.887
5665 measured reflections
2740 independent reflections
2435 reflections with *I* > 2σ(*I*)
*R*_int_ = 0.022

#### Refinement

*R*[*F* ^2^ > 2σ(*F* ^2^)] = 0.028
*wR*(*F* ^2^) = 0.064
*S* = 1.08
2740 reflections
154 parameters
H-atom parameters constrained
Δρ_max_ = 0.40 e Å^−3^
Δρ_min_ = −0.19 e Å^−3^
Absolute structure: Flack (1983 ▶), 1131 Friedel pairs
Flack parameter: 0.027 (13)

### 

Data collection: *SMART* (Bruker, 1997 ▶); cell refinement: *SAINT* (Bruker, 1997 ▶); data reduction: *SAINT*; program(s) used to solve structure: *SHELXS97* (Sheldrick, 2008 ▶); program(s) used to refine structure: *SHELXL97* (Sheldrick, 2008 ▶); molecular graphics: *SHELXTL* (Sheldrick, 2008 ▶); software used to prepare material for publication: *SHELXTL*.

## Supplementary Material

Crystal structure: contains datablocks I, global. DOI: 10.1107/S1600536809020042/bt2961sup1.cif

Structure factors: contains datablocks I. DOI: 10.1107/S1600536809020042/bt2961Isup2.hkl

Additional supplementary materials:  crystallographic information; 3D view; checkCIF report

## Figures and Tables

**Table 1:** Hydrogen-bond geometry (Å, °)

*D*—H⋯*A*	*D*—H	H⋯*A*	*D*⋯*A*	*D*—H⋯*A*
N1—H1*C*⋯O4^i^	0.93	2.17	3.075 (2)	164
N2—H2*C*⋯O2^ii^	0.93	2.13	2.987 (2)	152
O3—H3*A*⋯O2^iii^	0.84	1.70	2.532 (2)	170

Symmetry codes: (i) 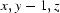; (ii) 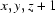; (iii) 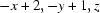.

**Fig. 1.**
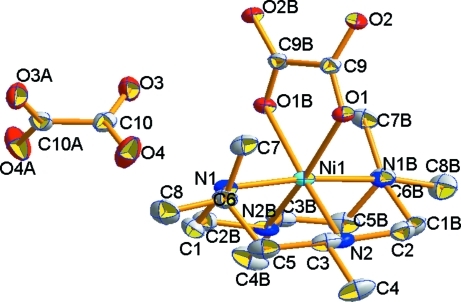
The molecular structure of the title compound, showing displacement ellipsoids at the 50% probability level; symmetry codes for the generated atoms: A(1 - x, 2 - y, z), B(2 - x, -y, z). H-atoms have been excluded for clarity.

**Fig. 2.**
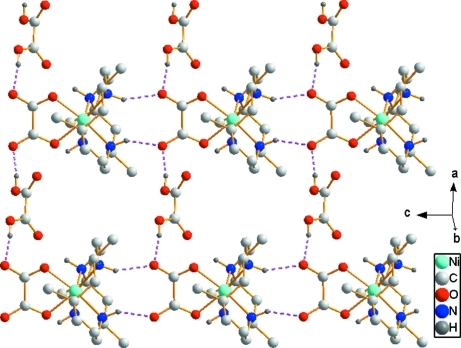
The hydrogen bond pattern in the title compound.

## supplementary crystallographic information

### Comment

Recently, many helical structures have been constructed through the coordination interactions of the organic ligand with suitable metal ions. Helical polymers constructed *via* hydrogen bonding, which is a versatile and efficient strategy, are still rare, and only a few cases have been reported. Then we employ chiral macrocyclic ligand *L* and oxalic acid as building blocks to construct helical structure, and unfortunately the helical structure is not obtained.

As illustrated in Fig.1, the six-coordinated Ni centre displays a distorted octahedral geometry. Neighbouring molecules are   connected through intermolecular N-H···O and O-H···O hydrogen bonds (Fig. 2).

### Experimental

Oxalic acid (0.5 g, 4 mmol) and NaOH (0.08 g, 2 mmol) were dissolved in 15 ml of water. To this solution was added [Ni(C_16_H_36_N_4_)](ClO_4_)_2_ (0.54 g, 1 mmol) dissolved in 2 ml of CH_3_CN. The solution was left to stand at room temperature and violet crystals formed after several weeks.

### Refinement

All H atoms were placed in calculated positions (O—H = 0.84Å, N—H = 0.93Å, C—H 0.98 to 1.00 Å) and were included in the refinement in the riding model approximation, with *U*_iso_ (H) set to 1.2 *U*_eq_(C,N) or 1.5 *U*_eq_(C_methyl_,O).

### Crystal data

**Table:**

[Ni(C_2_O_4_)(C_16_H_36_N_4_)]·C_2_H_2_O_4_	*F*(000) = 556
*M**_r_* = 521.25	*D*_x_ = 1.369 Mg m^−^^3^
Orthorhombic, *P*2_1_2_1_2	Mo *K*α radiation, λ = 0.71073 Å
Hall symbol: P 2 2ab	Cell parameters from 3630 reflections
*a* = 10.1261 (15) Å	θ = 2.4–26.9°
*b* = 15.515 (2) Å	µ = 0.82 mm^−^^1^
*c* = 8.0467 (11) Å	*T* = 173 K
*V* = 1264.2 (3) Å^3^	Prism, violet
*Z* = 2	0.48 × 0.21 × 0.15 mm

### Data collection

**Table:**

Bruker SMART CCD area-detector diffractometer	2740 independent reflections
Radiation source: fine-focus sealed tube	2435 reflections with *I* > 2σ(*I*)
graphite	*R*_int_ = 0.022
φ and ω scans	θ_max_ = 27.1°, θ_min_ = 2.4°
Absorption correction: multi-scan (*SADABS*; Sheldrick, 1996)	*h* = −12→12
*T*_min_ = 0.695, *T*_max_ = 0.887	*k* = −19→8
5665 measured reflections	*l* = −10→8

### Refinement

**Table:**

Refinement on *F*^2^	Secondary atom site location: difference Fourier map
Least-squares matrix: full	Hydrogen site location: inferred from neighbouring sites
*R*[*F*^2^ > 2σ(*F*^2^)] = 0.028	H-atom parameters constrained
*wR*(*F*^2^) = 0.064	*w* = 1/[σ^2^(*F*_o_^2^) + (0.0267*P*)^2^] where *P* = (*F*_o_^2^ + 2*F*_c_^2^)/3
*S* = 1.08	(Δ/σ)_max_ = 0.001
2740 reflections	Δρ_max_ = 0.40 e Å^−^^3^
154 parameters	Δρ_min_ = −0.19 e Å^−^^3^
0 restraints	Absolute structure: Flack (1983), 1131 Friedel pairs
Primary atom site location: structure-invariant direct methods	Flack parameter: 0.027 (13)

### Special details

**Table:**

Geometry. All e.s.d.'s (except the e.s.d. in the dihedral angle between two l.s. planes) are estimated using the full covariance matrix. The cell e.s.d.'s are taken into account individually in the estimation of e.s.d.'s in distances, angles and torsion angles; correlations between e.s.d.'s in cell parameters are only used when they are defined by crystal symmetry. An approximate (isotropic) treatment of cell e.s.d.'s is used for estimating e.s.d.'s involving l.s. planes.
Refinement. Refinement of *F*^2^ against ALL reflections. The weighted *R*-factor *wR* and goodness of fit *S* are based on *F*^2^, conventional *R*-factors *R* are based on *F*, with *F* set to zero for negative *F*^2^. The threshold expression of *F*^2^ > σ(*F*^2^) is used only for calculating *R*-factors(gt) *etc*. and is not relevant to the choice of reflections for refinement. *R*-factors based on *F*^2^ are statistically about twice as large as those based on *F*, and *R*- factors based on ALL data will be even larger.

### Fractional atomic coordinates and isotropic or equivalent isotropic displacement parameters (Å^2^)

**Table:**

	*x*	*y*	*z*	*U*_iso_*/*U*_eq_	
Ni1	1.0000	0.0000	0.60729 (3)	0.02079 (9)	
C10	0.57461 (18)	1.00225 (19)	0.2451 (2)	0.0304 (4)	
O1	1.09484 (13)	0.05954 (8)	0.40383 (17)	0.0245 (3)	
N1	0.81963 (17)	0.07108 (10)	0.6220 (2)	0.0256 (4)	
H1C	0.7759	0.0597	0.5227	0.031*	
C3	1.0605 (3)	0.17864 (15)	0.7602 (3)	0.0338 (6)	
H3	1.0887	0.1991	0.6478	0.041*	
C5	0.9146 (3)	0.19508 (15)	0.7775 (3)	0.0366 (6)	
H5A	0.9025	0.2577	0.7950	0.044*	
H5B	0.8846	0.1659	0.8801	0.044*	
N2	1.09305 (18)	0.08487 (10)	0.7733 (2)	0.0271 (4)	
H2C	1.0718	0.0672	0.8804	0.032*	
O2	1.12362 (14)	0.03860 (10)	0.13153 (16)	0.0360 (4)	
O3	0.62920 (15)	0.94107 (11)	0.1583 (2)	0.0423 (4)	
H3A	0.7117	0.9467	0.1609	0.064*	
O4	0.63195 (17)	1.05745 (11)	0.3227 (2)	0.0520 (5)	
C2	1.2350 (2)	0.06808 (14)	0.7481 (3)	0.0331 (5)	
H2A	1.2869	0.0964	0.8369	0.040*	
H2B	1.2636	0.0920	0.6399	0.040*	
C9	1.0624 (2)	0.02855 (12)	0.2656 (2)	0.0249 (4)	
C1	0.7410 (2)	0.02740 (14)	0.7515 (3)	0.0334 (5)	
H1A	0.6460	0.0390	0.7329	0.040*	
H1B	0.7652	0.0505	0.8620	0.040*	
C6	0.8215 (2)	0.16770 (13)	0.6365 (2)	0.0301 (5)	
C4	1.1353 (3)	0.23189 (14)	0.8912 (3)	0.0486 (7)	
H4A	1.1131	0.2930	0.8782	0.073*	
H4B	1.2306	0.2240	0.8764	0.073*	
H4C	1.1097	0.2126	1.0027	0.073*	
C8	0.6827 (3)	0.20270 (16)	0.6697 (3)	0.0452 (6)	
H8A	0.6209	0.1782	0.5888	0.068*	
H8B	0.6833	0.2656	0.6594	0.068*	
H8C	0.6551	0.1866	0.7823	0.068*	
C7	0.8682 (2)	0.20360 (14)	0.4693 (3)	0.0343 (5)	
H7A	0.9557	0.1803	0.4432	0.051*	
H7B	0.8731	0.2666	0.4756	0.051*	
H7C	0.8057	0.1869	0.3821	0.051*	

### Atomic displacement parameters (Å^2^)

**Table:**

	*U*^11^	*U*^22^	*U*^33^	*U*^12^	*U*^13^	*U*^23^
Ni1	0.02928 (17)	0.02073 (15)	0.01236 (14)	−0.00015 (18)	0.000	0.000
C10	0.0352 (10)	0.0320 (10)	0.0240 (9)	−0.0018 (13)	−0.0030 (8)	0.0069 (14)
O1	0.0311 (7)	0.0263 (7)	0.0160 (7)	0.0006 (6)	−0.0020 (6)	0.0021 (6)
N1	0.0341 (9)	0.0269 (8)	0.0159 (8)	0.0017 (7)	0.0010 (8)	−0.0023 (7)
C3	0.0562 (15)	0.0247 (11)	0.0205 (11)	−0.0054 (11)	−0.0064 (11)	−0.0006 (9)
C5	0.0602 (18)	0.0221 (11)	0.0277 (13)	0.0046 (11)	−0.0021 (12)	−0.0058 (10)
N2	0.0421 (11)	0.0249 (9)	0.0142 (8)	−0.0023 (8)	−0.0032 (7)	0.0006 (7)
O2	0.0294 (8)	0.0628 (10)	0.0158 (7)	−0.0010 (7)	0.0028 (6)	0.0070 (7)
O3	0.0281 (8)	0.0581 (11)	0.0408 (9)	−0.0047 (8)	0.0026 (7)	−0.0161 (8)
O4	0.0439 (10)	0.0474 (10)	0.0647 (12)	0.0033 (8)	−0.0190 (9)	−0.0187 (9)
C2	0.0385 (13)	0.0368 (13)	0.0239 (11)	−0.0086 (11)	−0.0034 (10)	−0.0029 (10)
C9	0.0278 (10)	0.0297 (10)	0.0170 (9)	0.0073 (8)	−0.0008 (8)	0.0048 (8)
C1	0.0356 (12)	0.0391 (13)	0.0255 (11)	0.0041 (10)	0.0104 (10)	−0.0003 (9)
C6	0.0404 (13)	0.0262 (10)	0.0238 (11)	0.0077 (9)	−0.0002 (10)	−0.0034 (9)
C4	0.0816 (19)	0.0296 (11)	0.0346 (13)	−0.0077 (12)	−0.0185 (15)	−0.0059 (12)
C8	0.0556 (16)	0.0412 (13)	0.0388 (13)	0.0170 (12)	0.0040 (12)	−0.0066 (11)
C7	0.0477 (14)	0.0283 (11)	0.0269 (11)	0.0057 (10)	−0.0066 (11)	0.0049 (9)

### Geometric parameters (Å, °)

**Table:**

Ni1—N2	2.0990 (17)	N2—H2C	0.9300
Ni1—N2^i^	2.0990 (17)	O2—C9	1.254 (2)
Ni1—O1^i^	2.1110 (13)	O3—H3A	0.8400
Ni1—O1	2.1110 (13)	C2—C1^i^	1.501 (3)
Ni1—N1	2.1368 (16)	C2—H2A	0.9900
Ni1—N1^i^	2.1368 (16)	C2—H2B	0.9900
C10—O4	1.209 (3)	C9—C9^i^	1.543 (4)
C10—O3	1.302 (3)	C1—C2^i^	1.501 (3)
C10—C10^ii^	1.513 (4)	C1—H1A	0.9900
O1—C9	1.255 (2)	C1—H1B	0.9900
N1—C1	1.477 (3)	C6—C8	1.530 (3)
N1—C6	1.504 (2)	C6—C7	1.531 (3)
N1—H1C	0.9300	C4—H4A	0.9800
C3—N2	1.495 (3)	C4—H4B	0.9800
C3—C5	1.506 (3)	C4—H4C	0.9800
C3—C4	1.539 (3)	C8—H8A	0.9800
C3—H3	1.0000	C8—H8B	0.9800
C5—C6	1.535 (3)	C8—H8C	0.9800
C5—H5A	0.9900	C7—H7A	0.9800
C5—H5B	0.9900	C7—H7B	0.9800
N2—C2	1.475 (3)	C7—H7C	0.9800
			
N2—Ni1—N2^i^	100.97 (9)	Ni1—N2—H2C	107.5
N2—Ni1—O1^i^	166.48 (6)	C10—O3—H3A	109.5
N2^i^—Ni1—O1^i^	90.84 (6)	N2—C2—C1^i^	109.25 (19)
N2—Ni1—O1	90.84 (6)	N2—C2—H2A	109.8
N2^i^—Ni1—O1	166.48 (6)	C1^i^—C2—H2A	109.8
O1^i^—Ni1—O1	78.29 (7)	N2—C2—H2B	109.8
N2—Ni1—N1	91.42 (7)	C1^i^—C2—H2B	109.8
N2^i^—Ni1—N1	84.55 (6)	H2A—C2—H2B	108.3
O1^i^—Ni1—N1	83.10 (6)	O2—C9—O1	125.80 (19)
O1—Ni1—N1	101.88 (5)	O2—C9—C9^i^	118.43 (12)
N2—Ni1—N1^i^	84.55 (6)	O1—C9—C9^i^	115.74 (11)
N2^i^—Ni1—N1^i^	91.42 (7)	N1—C1—C2^i^	110.65 (19)
O1^i^—Ni1—N1^i^	101.88 (5)	N1—C1—H1A	109.5
O1—Ni1—N1^i^	83.10 (6)	C2^i^—C1—H1A	109.5
N1—Ni1—N1^i^	173.67 (9)	N1—C1—H1B	109.5
O4—C10—O3	126.15 (19)	C2^i^—C1—H1B	109.5
O4—C10—C10^ii^	120.8 (3)	H1A—C1—H1B	108.1
O3—C10—C10^ii^	113.0 (3)	N1—C6—C8	110.86 (18)
C9—O1—Ni1	113.59 (12)	N1—C6—C7	107.34 (15)
C1—N1—C6	114.16 (15)	C8—C6—C7	107.94 (18)
C1—N1—Ni1	105.25 (12)	N1—C6—C5	109.95 (17)
C6—N1—Ni1	120.54 (13)	C8—C6—C5	109.69 (18)
C1—N1—H1C	105.2	C7—C6—C5	111.02 (19)
C6—N1—H1C	105.2	C3—C4—H4A	109.5
Ni1—N1—H1C	105.2	C3—C4—H4B	109.5
N2—C3—C5	112.0 (2)	H4A—C4—H4B	109.5
N2—C3—C4	111.46 (19)	C3—C4—H4C	109.5
C5—C3—C4	109.2 (2)	H4A—C4—H4C	109.5
N2—C3—H3	108.0	H4B—C4—H4C	109.5
C5—C3—H3	108.0	C6—C8—H8A	109.5
C4—C3—H3	108.0	C6—C8—H8B	109.5
C3—C5—C6	119.2 (2)	H8A—C8—H8B	109.5
C3—C5—H5A	107.5	C6—C8—H8C	109.5
C6—C5—H5A	107.5	H8A—C8—H8C	109.5
C3—C5—H5B	107.5	H8B—C8—H8C	109.5
C6—C5—H5B	107.5	C6—C7—H7A	109.5
H5A—C5—H5B	107.0	C6—C7—H7B	109.5
C2—N2—C3	112.13 (17)	H7A—C7—H7B	109.5
C2—N2—Ni1	103.84 (12)	C6—C7—H7C	109.5
C3—N2—Ni1	117.82 (13)	H7A—C7—H7C	109.5
C2—N2—H2C	107.5	H7B—C7—H7C	109.5
C3—N2—H2C	107.5		
			
N2—Ni1—O1—C9	−177.81 (13)	O1—Ni1—N2—C2	−60.63 (12)
N2^i^—Ni1—O1—C9	31.2 (3)	N1—Ni1—N2—C2	−162.53 (13)
O1^i^—Ni1—O1—C9	−5.92 (10)	N1^i^—Ni1—N2—C2	22.36 (12)
N1—Ni1—O1—C9	−86.19 (13)	N2^i^—Ni1—N2—C3	−122.59 (18)
N1^i^—Ni1—O1—C9	97.78 (13)	O1^i^—Ni1—N2—C3	27.8 (4)
N2—Ni1—N1—C1	−94.43 (13)	O1—Ni1—N2—C3	64.03 (16)
N2^i^—Ni1—N1—C1	6.46 (13)	N1—Ni1—N2—C3	−37.87 (16)
O1^i^—Ni1—N1—C1	97.97 (13)	N1^i^—Ni1—N2—C3	147.02 (16)
O1—Ni1—N1—C1	174.42 (12)	C3—N2—C2—C1^i^	−176.24 (19)
N1^i^—Ni1—N1—C1	−44.13 (12)	Ni1—N2—C2—C1^i^	−48.0 (2)
N2—Ni1—N1—C6	36.38 (14)	Ni1—O1—C9—O2	−163.44 (16)
N2^i^—Ni1—N1—C6	137.26 (14)	Ni1—O1—C9—C9^i^	14.9 (2)
O1^i^—Ni1—N1—C6	−131.22 (14)	C6—N1—C1—C2^i^	−169.19 (19)
O1—Ni1—N1—C6	−54.78 (14)	Ni1—N1—C1—C2^i^	−34.8 (2)
N1^i^—Ni1—N1—C6	86.67 (13)	C1—N1—C6—C8	−45.1 (2)
N2—C3—C5—C6	−70.9 (3)	Ni1—N1—C6—C8	−171.97 (14)
C4—C3—C5—C6	165.16 (19)	C1—N1—C6—C7	−162.80 (18)
C5—C3—N2—C2	177.2 (2)	Ni1—N1—C6—C7	70.36 (19)
C4—C3—N2—C2	−60.2 (2)	C1—N1—C6—C5	76.3 (2)
C5—C3—N2—Ni1	56.7 (2)	Ni1—N1—C6—C5	−50.5 (2)
C4—C3—N2—Ni1	179.33 (16)	C3—C5—C6—N1	66.3 (3)
N2^i^—Ni1—N2—C2	112.75 (13)	C3—C5—C6—C8	−171.6 (2)
O1^i^—Ni1—N2—C2	−96.8 (3)	C3—C5—C6—C7	−52.4 (3)

Symmetry codes: (i) −*x*+2, −*y*, *z*; (ii) −*x*+1, −*y*+2, *z*.

### Hydrogen-bond geometry (Å, °)

**Table:**

*D*—H···*A*	*D*—H	H···*A*	*D*···*A*	*D*—H···*A*
N1—H1C···O4^iii^	0.93	2.17	3.075 (2)	164
N2—H2C···O2^iv^	0.93	2.13	2.987 (2)	152
O3—H3A···O2^v^	0.84	1.70	2.532 (2)	170

Symmetry codes: (iii) *x*, *y*−1, *z*; (iv) *x*, *y*, *z*+1; (v) −*x*+2, −*y*+1, *z*.
